# Hypoxic ADSCs-derived EVs promote the proliferation and chondrogenic differentiation of cartilage stem/progenitor cells

**DOI:** 10.1080/21623945.2021.1945210

**Published:** 2021-07-05

**Authors:** Ke Xue, Yongkang Jiang, Xiaodie Zhang, Jun Wu, Lin Qi, Kai Liu

**Affiliations:** a Department of Plastic and Reconstructive Surgery, Shanghai 9th People’s Hospital, Shanghai Jiao Tong University School of Medicine, Shanghai Key Laboratory of Tissue Engineering, Shanghai, PR China; b Department of Orthopedics, The First People’s Hospital of Changzhou, Jiangsu Changzhou, China; c Department of Radiology, Huadong Hospital, Fudan University, Shanghai, China

**Keywords:** Cartilage stem/progenitor cells, extracellular vesicles, hypoxic adipose-derived stem cells, cartilage tissue engineering, proliferation, differentiation

## Abstract

Cartilage tissue engineering is a promising option for repairing cartilage defects, although harvesting a large number of seeding cells remains a major challenge. Cartilage stem/progenitor cells (CSPCs) seem to be a promising cell source. Hypoxic extracellular vesicles (EVs) may play a major role in cell-cell and tissue-tissue communication. In the current study, we aimed to evaluate the effect of hypoxic adipose-derived stem cells (ADSCs)-derived EVs on CSPCs proliferation and differentiation. The characteristics of ADSCs-derived EVs were identified, and proliferation, migration, and cartilage-related gene expression of CSPCs were measured with or without the presence of hypoxic ADSCs-derived EVs. SEM, histological staining, biochemical and biomechanical analysis was performed to evaluate the effect of hypoxic ADSCs-derived EVs on CSPCs in alginate hydrogel culture. The results indicated that the majority of ADSC-derived EVs exhibited a round-shaped or cup-shaped morphology with a diameter of 40–1000 nm and expressed CD9, CD63, and CD81. CSPCs migration and proliferation were enhanced by hypoxic ADSCs-derived EVs, which also increased the expression of cartilage-related genes. The hypoxic ADSCs-derived EVs induce CSPCs to produce significantly more cartilage matrix and proteoglycan. In conclusion, hypoxic ADSCs-derived EVs improved the proliferation and chondrogenic differentiation of CSPCs for cartilage tissue engineering.

## 1. Introduction

Articular, auricular and nasal cartilage defects occur due to trauma, disease, and tumours [1,2]. However, injured cartilage tissue cannot repair and regenerate spontaneously, which may lead to structural abnormality and malfunction [3]. Current surgical methods include abrasion arthroplasty, drilling, and microfracture, although these techniques do not achieve ideal results [4], and the repair of cartilage defects is still a challenge for clinical surgeons [5]. A variety of procedures are applied to repair cartilage damage, and progress and development in cell biology and biomaterial scaffolds have promoted the exploration and therapeutic application of cartilage tissue engineering or cartilage regenerative medicine in the repair of cartilage tissue defects [6]. Cartilage tissue engineering or cartilage regenerative medicine mainly involves the repair, regeneration and replacement of aged, damaged and dead cells/tissue, restoring normal tissue structure and function [5]. The main factors of cartilage engineering involve seeding cells, scaffolds and the microenvironment, while seeding cells play a vital role. Chondrocytes were used as the first choice in cartilage engineering, as chondrocytes are the only cells found in cartilage [7]. Vacanti et al. reported seeding chondrocytes on synthetic polymers to produce a new cartilage tissue [8]. Cao Y et al. demonstrated the transplantation of chondrocytes utilizing a polymer-cell construct to produce tissue-engineered cartilage in the shape of a human ear [9].

The clinical application of chondrocyte-based cartilage tissue engineering is limited because of the low proliferation and dedifferentiation of chondrocytes after a long in vitro expansion [10]. It is well known that chondrocytes undergo dedifferentiation when expanded in vitro. Dedifferentiated chondrocytes produced a non-specific mechanically inferior extracellular cartilage matrix, characterized by reduced collagen matrix and glycosaminoglycan (GAG) accumulation [11,12]. During long-term in vitro expansion to obtain a large number of differentiated chondrocytes, chondrocytes will undergo dedifferentiation characterized by decreased expression of cartilage-specific markers, such as type II, XI, IX collagens (COL-2, COL-11, COL-9) and aggrecan (ACAN), and increased expression of type I collagen (COL-1) and type X collagen (COL-10) [13]. Stem cells, especially adult Mesenchymal stromal cells, have recently emerged, providing a new cell source for cartilage tissue engineering and cartilage regenerative medicine. Due to the high proliferation, multi-differentiation ability, and ease of harvesting, mesenchymal stromal cell-based cartilage tissue engineering are considered a promising approach for cartilage regeneration [14]. Mesenchymal stromal cells (MSCs) originating from bone marrow and adipose tissue, muscles and other sources have been reported to differentiate into chondrocytes after stimulation by growth factors in certain biological environments, and they have been used in the repair of cartilage defects [15]. However, an increasing number of studies have found multiple risks that limit the successful clinical application of MSCs. Intrinsic risk factors including immunosuppression, cellular rejection and infusion toxicity, and iatrogenic tumour formation and extrinsic risk factors including those induced by human handling, such as culture conditions, cryopreservation and various other cell manipulations, remain major challenges [16,17]. In addition, abnormalities in cell phenotype, proliferation and differentiation ability, and undesired hypertrophy and ossification of neo-cartilage differentiated from stem cells remain unresolved [18].

Therefore, seeding cells play an important role in cartilage tissue engineering because the fate of implanted seeding cells is directly related to the success of tissue-engineered cartilage. Chondrogenic stem/progenitor stem cells derived from cartilage tissue were isolated and identified recently. In our previous study, we isolated cartilage stem/progenitor stem cells by a differential adhesion assay to fibronectin, and evaluated the stemness of the cartilage stem/progenitor stem cells, which may become ideal seeding cells in cartilage tissue engineering [19, 20].

Recently, several recent studies have suggested that extracellular vesicles or micro-vesicles (MVs) released from MSCs carry microRNA (miRNA), mRNA, and protein and play vital roles in tissue regeneration and repair. Some evidence has demonstrated that MSC-secreted extracellular vesicles (EVs), including exosomes (40–100 nm in diameter) and micro-vesicles (MVs; 0.1–1 mm in diameter), may mediate cell-cell micro-communication and transport paracrine factors during tissue repair, tissue regeneration and immune regulation to exert therapeutic effects [21–24]. Tao et al. demonstrated that miR-140-5p-overexpressing human synovial MSC-derived exosomes enhanced cartilage tissue regeneration and prevented osteoarthritis of the knee in a rat model [25]. Liu et al. also showed the integration of exosomes derived from hiPSC-derived MSCs with in situ hydrogel glue as a promising tissue patch for articular cartilage regeneration [26].

Zhang et al. demonstrated that exosomes derived from human embryonic Mesenchymal stromal cells promote osteochondral regeneration [27]. Stella Cosenza et al. reported that Mesenchymal stromal cells derived exosomes and microparticles protect cartilage and bone from degradation in osteoarthritis [28]. In addition, extracellular vesicles secreted by hypoxia pre-conditioned renal proximal tubular cells were reported to exert more effective therapeutic effects in tissue repair and tissue regeneration [29].

To date, there have been few reports on the effect of hypoxic extracellular vesicles on the proliferation and differentiation of cartilage stem/progenitor stem cells. In the present study, we aimed to evaluate the effectiveness of hypoxic extracellular vesicles isolated from adipose-derived stem cells (ADSCs) on cartilage stem/progenitor stem cells differentiation in 2D and 3D (alginate hydrogel culture) conditions and to explore the feasibility of hypoxic ADSC-derived EVs integrated with alginate hydrogel in cartilage tissue engineering. We found that hypoxic ADSC-derived EVs could stimulate cartilage stem/progenitor stem cells migration and proliferation and downregulate chondrocyte hypertrophic-related genes, suggesting its potential for use in cartilage tissue engineering and cartilage regeneration.

## 2. Method and materials

All experimental procedures in this study were approved by the Ethics Committee of Ninth People’s Hospital, Shanghai Jiao Tong University School of Medicine.

### 2.1 Cell harvest

The external ears of experimental rabbit were obtained and cut into small pieces as previously reported [30]. The isolated chondrocytes obtained from the auricular cartilage slices by digestion with (0.2% w/v) collagenase II were cultured in DMEM containing 10% foetal bovine serum (FBS), 100 U/ml penicillin, and 100 U/ml streptomycin. Then, the suspension was filtered through a 200-μm filter to remove undigested particles, and chondrocytes at a density of 4000 cells/ml were seeded onto 10-cm plastic dishes (treated with 10 μg/ml fibronectin overnight) at 37°C for 20 min in low-glucose DMEM. After 20 min, nonadherent cells and media were removed, and low-glucose DMEM containing 10% FBS was added to the plates. The adherent cells were cultured for 7–14 days until the cells reached 80–90% confluence. The cells were then digested with 0.25% trypsin plus 0.02% EDTA (Invitrogen) and sub-cultured into new dishes at a density of 2 × 10^4^ cells/cm^2^. In our previous study, flow cytometric analysis demonstrated the cell populations expressed mesenchyme stem cell positive surface marker and the cells differentiate into osteogenic line, chondrogenic line and adipogenic line under different induction conditions.

The fat tissues were harvested from subcutaneous adipose tissue, minced and digested with 0.075% collagenase type I in the shaking bed at 37°C for 40 min. The digested mixture was centrifuged at 1200 × g for 15 min. Then, the pellet was resuspended in low-glucose DMEM containing 10% foetal bovine serum (FBS) and 1% penicillin/streptomycin in a humidified atmosphere containing 95% air and 5% CO_2_ at 37°C. The medium was changed every 2 days until 80–90% confluency. In our previous study Flow cytometric analysis demonstrated the cell populations expressed mesenchyme stem cell positive surface marker and the cells differentiate into osteogenic line, chondrogenic line and adipogenic line under different induction conditions.

### 2.2 Isolation of *extracellular vesicles*

Before EV isolation, ADSCs at about 85% confluent were washed with complete culture medium and then cultured under 20% O_2_ (normoxic) OR 1% O_2_ (hypoxic) conditions for 24 h. The isolation of extracellular vesicles from normoxic ADSC medium or hypoxic medium followed the multistep ultracentrifugation process as previously reported [31]. Briefly, the conditioned medium containing extracellular vesicles was obtained by centrifugation at 2000 × g for 10 min and 10,000 × g for 30 min at 4°C to remove dead cells and cell debris, followed by filtering with a sterilized filter (0.22 μm, Millipore). The supernatant was further ultra-centrifuged at 110,000 × g for 90 min at 4°C. The EV at the bottom of the centrifuge tube was washed with phosphate-buffered saline (PBS) and stored at −80°C until use.

### 2.3 Characterization of ADSC-derived EVs

Particle size and number were measured on an Izon qNano system by TRPS technology (Izon Science, Ltd. Christchurch, New Zealand) as previously described [32]. Tween-20 (0.03% (w/w) in PBS) was used for all dilutions in the current experiment. The nanopore was stretched to 47.00 mm and then wetted with electrolyte under a pressure of 2000 Pa for one minute. The stretching was then optimized with an applied voltage of 0.96 V for calibration particles at 45.05 mm. Calibration was performed using carboxylated polystyrene nanoparticles under a pressure of 400 Pa at a dilution of 1/1000 (v/v). The extracellular vesicles were diluted to 1/1000 (v/v) and measured under a constant pressure of 400 Pa in triplicate. The particle size distribution was calculated using qNano software (Izon Control Suite v 3.2).

The morphology of ADSC-derived EVs was observed by transmission electron microscopy (TEM) as previously reported [33]. Briefly, the extracellular vesicles were fixed with 0.1% glutaraldehyde in PBS for 30 minutes, rinsed in deionized water and dried, stained in 1% osmium tetroxide in PBS for 30 minutes, washed in deionized water three times and dried under a pure nitrogen stream. The stained extracellular vesicles were observed by TEM (60 KV, Jeol JEM-1230, Tokyo, Japan).

Specific markers of ADSC-derived EVs (CD9, CD63, CD81) were evaluated by Western blotting as previously reported [34].

Flow cytometry was used to analyse several extracellular vesicles molecules after vesicle adsorption onto latex beads as previously reported [35]. In brief, 2 mg of ADSC-derived EVs were incubated with 2 mL of aldehyde/sulphate latex beads (4% w/v, 4 µm, A37304, Invitrogen, Thermo Fisher Scientific, USA) for 2 hours at room temperature, followed by 30 minutes of incubation in PBS containing 2% FBS. Extracellular vesicles-coated beads were incubated with primary mouse anti-human monoclonal antibodies (CD9, CD63, CD81) for 30 min at 4°C and then incubated with polyclonal rabbit anti-mouse secondary antibodies for 20 min at 4°C. Samples were analysed using a Beckman Coulter FC500 flow cytometer (Beckman Coulter, USA).

### 2.4 Preparation of the *cartilage stem/progenitor cell*-hydrogel composite

A 2% (w/v) sodium alginate (ALG) solution was produced by dissolving alginic acid sodium salt (low viscosity; Sigma) in deionized water, and the solution was sterilized with a 0.22 um filter (Millipore). The alginic acid sodium salt solution was blended with cartilage stem/progenitor cell suspension (50 million/mL) and extruded into 0.1 M CaCl_2_ solution dropwise by using a syringe with a 23 G needle and crosslinked for 10 min to obtain cartilage stem/progenitor cell-hydrogel beads. The cartilage stem/progenitor cell – hydrogel beads were washed with DMEM without FBS and incubated in DMEM containing 10% FBS at 37°C and 5% CO_2_.

The cartilage stem/progenitor cell-hydrogel beads were incubated in with chondrogenic induction medium [serum-free α-MEM supplemented with ITS+ (GIBCO), 10 ng/mL TGF-β1 (RD), 10^−7^ M dexamethasone (Sigma), and 50 μg/mL/mL ascobate-2-phosphate (Sigma)], with hypoxic extracellular vesicles (50 μg/mL) for 3 weeks, the above medium was changed every other day.

In the control groups, cartilage stem/progenitor cell-hydrogel beads were incubated in with chondrogenic induction medium with or without normoxic extracellular vesicles (50 μg/mL) for 3 weeks, the above medium was changed every other day.

The specimens were harvested at 3 weeks and were sent for histological and immune-histological examination.

### 2.5 Real-time quantitative polymerase chain reaction

RT-PCR was used to assess some chondrocyte-related genes in cartilage stem/progenitor cells under hypoxia and hypoxic extracellular vesicles. Before cell harvesting, cartilage stem/progenitor cells at about 85% confluent were washed with complete culture medium and then cultured under 20% O_2_ (normoxic) or 1% O_2_ (hypoxic) conditions with chondrogenic induction medium [serum-free α-MEM supplemented with ITS+ (GIBCO), 10 ng/mL TGF-β1 (RD), 10^−7^ M dexamethasone (Sigma), and 50 μg/mL ascobate-2-phosphate (Sigma)]for 7 days.

For 3D culture, the cartilage stem/progenitor cell-hydrogel beads were incubated in chondrogenic induction medium [serum-free α-MEM supplemented with ITS+ (GIBCO), 10 ng/mL TGF-β1 (RD), 10^−7^ M dexamethasone (Sigma), and 50 μg/mL ascobate-2-phosphate (Sigma)] for 3 weeks at 37°C and 5% CO_2_, with or without hypoxic extracellular vesicles (50 μg/mL).

Total RNA was extracted from each specimen, and cDNA was obtained by reverse transcription (RT) according to previously described methods [36]. Real-time quantitative RT-PCR was used to evaluate the expression of the following cartilage-specific genes: type II collagen (COL II) a1, Sox-9, aggrecan, and hypertrophic genes: RUNX2, type X collagen (COL X) a1, and MMP13. The primers used in this study are shown in Table 1. The GAPDH mRNA level was quantified as an internal control.

**Table 1.** mRNASequence (5ʹ->3ʹ) Product length

COL2A1	CTGTGTCTGTGACACGGGGA	385
	TTGGGCAGCAAAGTTTCCAC	
Aggrecan	TACCTTGGAGGTCGTGGTGA	212
	TGTGGATGGGGTACCTGACA	
SOX9	TGAATCTCCTGGACCCCTTCAT	211
	ACGGGGAACTTGTCCTCTTC	
RUNX2	GACACTGCCACCTCTGACTT	213
	GGACATACCGAGGGACATGC	
COL10A1	CCAGGCCTGAAGGGACAAAT	187
	GCCCGATCTCACCTTTAGGG	
MMP13	ACAAACCACACTTGGGAGGG	171
	CTGCATCTTGAATGGCCGTG	
GAPDH	AGACACGATGGTGAAGGTCG	164
	TGCCGTGGGTGGAATCATAC	

### 2.6 Western blotting

ADSC-derived EVs were suspended in protein loading buffer and heated at 95°C for 5 min, followed by separation on 10% SDS-PAGE polyacrylamide gels and transfer to polyvinylidene fluoride (PVDF) membranes. Mouse anti-human CD9 (1:500, CST, USA), anti-CD81 (1:200, CST, USA), anti-CD63 (1:200, CST, USA), were used as the primary antibodies. Goat anti-mouse IgG-HRP (CST, USA) was used as the secondary antibody. The protein expression of MMP-13, collagen II and collagen X of the cartilage stem/progenitor cell-hydrogel beads at 3 weeks were evaluated by western blot according the method described above. The chemiluminescence signal was detected with enhanced chemiluminescence (Thermo) and imaged by Image Quant LAS 4000 mini bio-molecular imager (GE Healthcare, Uppsala, Sweden). The relative signal intensity was measured by ImageJ and normalized to controls as indicated.

### 2.7 Proliferation and migration of *cartilage stem/progenitor cells under hypoxia*

In general, tissue repair requires migration and proliferation, while cartilage stem/progenitor cells are embedded in extracellular matrix, which renders them unable to migrate to the injury site and proliferate to repair tissue defects. The cartilage stem/progenitor cells at passage 3 were seeded into each well of 96-well plates at 1 × 10^3^ cells for 24 h, were cultured under 20% O_2_ (normoxic) or 1% O_2_ (hypoxic) conditions, then proliferation of cartilage stem/progenitor cells was evaluated by a Cell Counting Kit-8 (CCK-8; Dojindo, Japan) as described previously [37]. The optical density (OD) value was determined at a wavelength of 450 nm by a microplate reader.

The scratch wound assay was used to analyse the effect of hypoxia on the migration of cartilage stem/progenitor cells as reported previously [38]. Briefly, 1.5 × 10^4^ cells were seeded into 12-well plates for 8 h, followed by scratching with a 200 P pipet tip, then were cultured under 20% O_2_ (normoxic) or 1% O_2_ (hypoxic) conditions, the scratch gap was recorded by collecting digital images at different time points (0 and 24 h) at the same site. The gap was measured using ImageJ software.

*2.8 Proliferation and migration of* cartilage stem/progenitor cells under hypoxic extracellular vesicles

In the current study, the effect of ADSC-derived EVs on the migration and proliferation of cartilage stem/progenitor cells was evaluated. The cartilage stem/progenitor cells at passage 3 were seeded into each well of 96-well plates at 1 × 10^3^ cells for 24 h, and then the culture medium was replaced by hypoxic extracellular vesicles or normoxic extracellular vesicles (50 μg/mL), a Cell Counting Kit-8 (CCK-8; Dojindo, Japan) was used to evaluate the effect of ADSC extracellular vesicles on cartilage stem/progenitor cells proliferation. The optical density (OD) value was determined at a wavelength of 450 nm by a microplate reader.

The scratch wound assay was used to analyse the effect of ADSC extracellular vesicles on the migration of cartilage stem/progenitor cells. Briefly, 1.5 × 10^4^ cells were seeded into 12-well plates for 8 h, followed by scratching with a 200 P pipet tip. The medium was removed, and the cells were washed once with PBS. Then, the medium was replaced with fresh DMEM hypoxic extracellular vesicles or normoxic extracellular vesicles (50 μg/mL). The scratch gap was recorded by collecting digital images at different time points (0 and 24 h) at the same site. The gap was measured using ImageJ software.

### 2.9 Cell viability assessment

To study the cell viability, the cartilage stem/progenitor cell-hydrogel construct harvested at 3 weeks was rinsed twice in PBS, and then stained with a Live/Dead staining kit (Invitrogen) according to the manufacturer’s instructions. Fluorescent photo-micrographic images of stained cells in the hydrogels were acquired using a fluorescence microscope equipped with a digital camera (DMI 3000, Leica).

### 2.10 Scanning electron microscopy (SEM)

The hydrogel scaffolds and cartilage stem/progenitor cell-hydrogel constructs at 3 weeks were collected and fixed in 4% paraformaldehyde/0.1 M sodium cacodylate buffer containing 10 mM CaCl_2_ (pH 7.4) for 24 h at 4°C. The beads were dehydrated in a series of ethanol concentrations (40–100%). The SEM (Hitachi S-4800, Japan) was used to examine the surface and morphologies of hydrogel scaffolds, as well as chondrocytes-hydrogel constructs.

### 2.11 Analysis of in vitro chondrogenesis

The hydrogels combined with cartilage stem/progenitor cell were harvested at 3 weeks, specimens were fixed in buffered 10% formalin in PBS for 24 h and dehydrated in a series of ethanol concentrations. Afterwards, the beads were embedded in paraffin and sectioned (5-um thickness). The sections were stained with haematoxylin and eosin (HE), Safranin O, and collagen II staining (1:100 in PBS, Santa Cruz Biotechnology, Santa Cruz, CA, USA) to evaluate histological structure and cartilage matrix deposition in cartilage stem/progenitor cell-hydrogel constructs.

After 3 weeks of in vitro culture, glycosaminoglycan (GAG) content and total collagen content were analysed. GAG content was evaluated by a modified 1,9 dimethylmethylene blue (DMMB) dye-binding assay with chondroitin-6-sulphate (Sigma), as previously described [39]. To account for the anionic nature of the carboxyl groups on the alginate hydrogel, the pH of the DMMB dye was adjusted to 1.5 with concentrated formic acid (Sigma) so that only the sulphated GAG-DMB complexes were detectable at a wavelength of between 540 and 595 nm by a spectrophotometer.

The collagen content was determined as previously described [40]. Briefly, the sample was hydrolysed with 2.0 M sodium hydroxide for 20 min at 120°C. Then, the hydrolyzate was oxidized using a buffered chloramine-T reagent (Sigma) for 25 min followed by the addition of Ehrlich’s reagent. Absorbance was measured at 550 nm. The hydroxyproline content was obtained by interpolation along a standard curve, and the obtained hydroxyproline content was converted to collagen content using a 1:10 conversion ratio of hydroxyproline to collagen [41].

The hydrogels combined with cartilage stem/progenitor cell were harvested at 3 weeks and sent for biomechanical testing, and the compressive modulus of the samples was calculated based on the force displacement curve.

### 2.12 Statistical analysis

All data were analysed and expressed as the mean ± SD for three experimental groups (n = 6). The effects of various parameters on the characteristics of the developed hydrogels were statistically analysed by one-way analysis of variance. A p-value less than 0.05 was considered statistically significant.

## 3. Results

### 3.1 *Hypoxia* promoted the migration and proliferation of *cartilage stem/progenitor cell*

**Figure 1.**
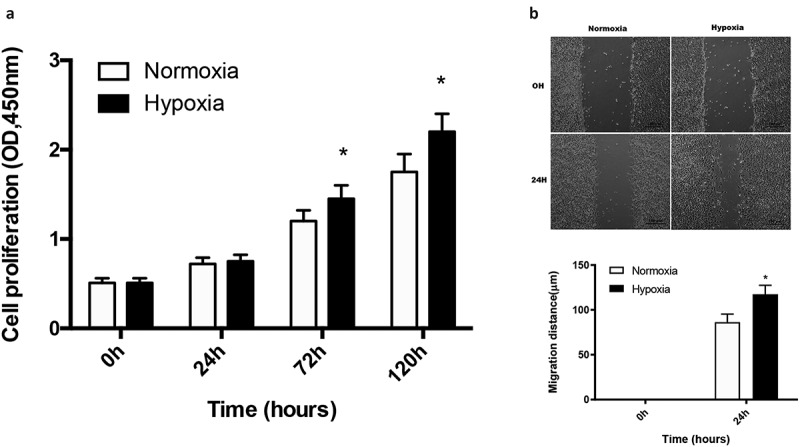
Hypoxia promoted the migration and proliferation of cartilage stem/progenitor cell

Cartilage stem/progenitor cells were cultured in different concentrations of O_2_ (20% O_2_ or 1% O_2_) for 5 days. As shown in Figure 1, the cartilage stem/progenitor cell proliferated with increasing culture period, and there was an obvious difference in cell proliferation between the normoxic group and the hypoxic group (P < 0.05), which indicated that hypoxia may promote cell proliferation.

The cartilage stem/progenitor cell cultured in different concentrations of O_2_ (20% O_2_ or 1% O_2_) proliferated with increasing time in culture, while the hypoxic group (1% O_2_) show a higher cell proliferation than the normoxic group (P < 0.05). There was an obvious difference in cell migration between the normoxic group and the hypoxic group (P < 0.05) in the scratch wound assays, which indicated that hypoxia may promote cell motility.

Scratch wound assays indicated that 1% O_2_ significantly enhanced the motility of cartilage stem/progenitor cell (P < 0.05), indicating that hypoxia enhanced cartilage stem/progenitor cell migration (Figure 1).

### 3.2 Hypoxia *promoted the chondrogenic differentiation and inhibit the terminal differentiation of* cartilage stem/progenitor cell

**Figure 2.**
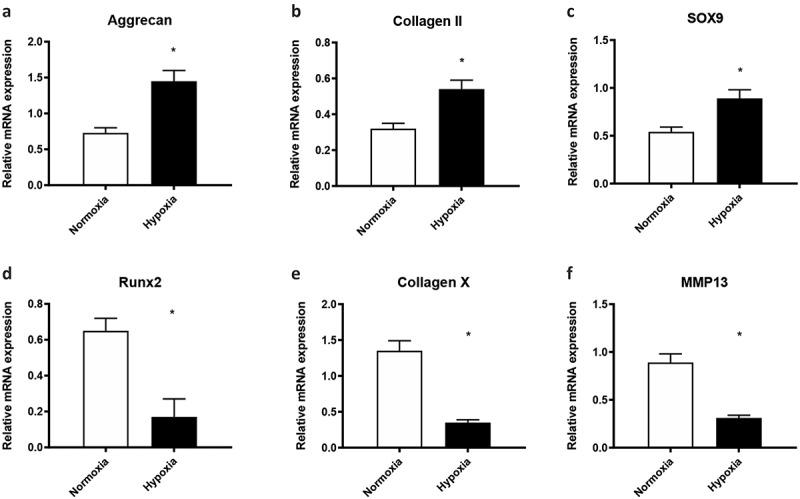
Hypoxia promoted the chondrogenic differentiation and prevents the terminal differentiation of cartilage stem/progenitor cell

Hypoxia was reported to promote the chondrogenic differentiation of BMSCs, while there is no report on the effect of hypoxia on the chondrogenic differentiation of cartilage stem/progenitor cell. When cultured in 1% O_2_, the expression of aggrecan, type II collagen, and Sox-9 was significantly upregulated (p < 0.05) on day 7 of induction, while the expression of these chondrocyte-specific genes in the control group remained very low, which suggested that hypoxia promoted the differentiation of cartilage stem/progenitor cell (Figure 2). By contrast, 1% O_2_ decreased the mRNA levels of Runx2 and Col10a1, as well as MMP13 compared to 20% O_2_ (p < 0.01), that are the markers of hypertrophic genes or endochondral ossification in chondrocytes.

1% O_2_ upregulated the mRNA levels of aggrecan, type II collagen, and Sox-9 compared to the expression of these chondrocyte-specific genes cultured in 20% O_2_ (p < 0.01) on day 7 of induction. The expression of Runx2 and Col10a1, as well as MMP13 of cartilage stem/progenitor cell cultured in 1% O_2_ was significantly downregulated (p < 0.05), compared with these in 20% O_2_ group on day 7 of induction.

### 3.3 Characterization of ADSC-derived EVs

**Figure 3.**
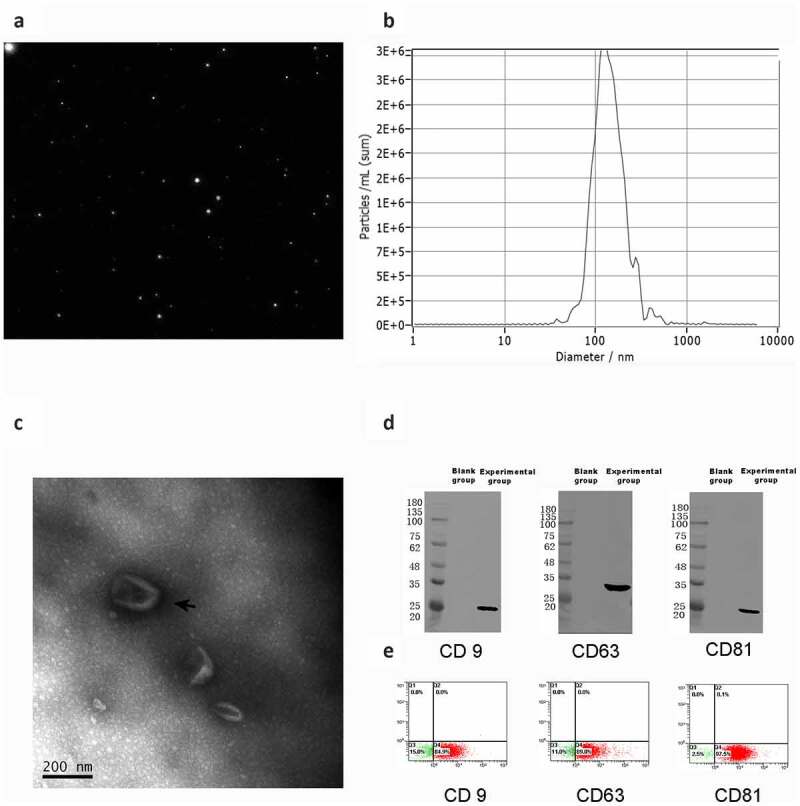
**Characterization of ADSC-derived EVs** (a-b) The size distribution of ADSC extracellular vesicles was approximately 40 to 1000 nm by particle size analysis. (c) TEM indicated that ADSC extracellular vesicles exhibited a round-shaped or cup-shaped morphology. (d) Western blotting analyses confirmed the presence of extracellular vesicles marker proteins, such as CD9, CD63, and CD81 proteins. (e) High expression of the three extracellular vesicles markers (CD9, CD63 and CD81) was revealed on the ADSC-derived EVs by cytofluorimetric assay

The size and number of ADSC extracellular vesicles were analysed, and the size distribution was approximately 40 to 1000 nm. In addition, TEM indicated that ADSC extracellular vesicles exhibited a round-shaped or cup-shaped morphology with a diameter of 40–1000 nm. Western blotting analyses confirmed the presence of extracellular vesicles marker proteins, such as CD9, CD63, and CD81 proteins. High expression of the three extracellular vesicles markers (CD9, CD63 and CD81) was revealed on the ADSC-derived EVs by cytofluorimetric assay (Figure 3).

### 3.4 Hypoxic ADSCs-derived EVs enhanced the migration and proliferation of cartilage stem/progenitor cell

**Figure 4.**
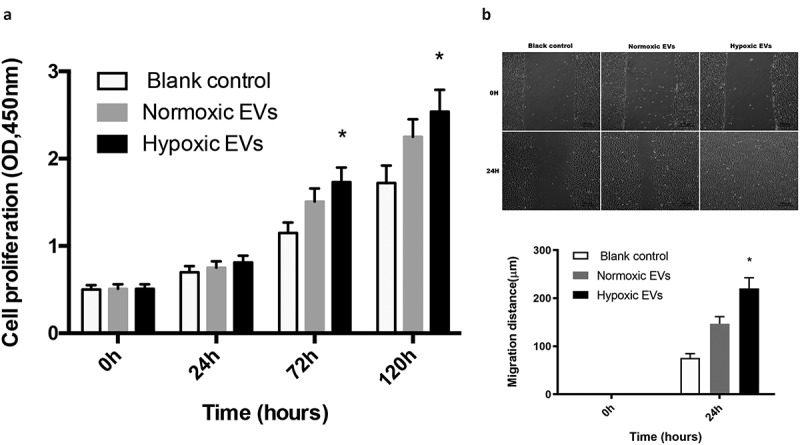
**Hypoxic ADSCs-derived EVs enhanced the migration and proliferation of cartilage stem/progenitor cell** (a) Cartilage stem/progenitor cell were cultured in different condition of ADSC extracellular vesicles for 7 days, and the hypoxic ADSCs-derived EVs may promote cell proliferation significantly, compared with the normoxic ADSCs-derived EVs group (P < 0.05). (b) Hypoxic ADSCs-derived EVs significantly enhanced the motility of cartilage stem/progenitor cell in scratch wound assays (P < 0.05)

Cartilage stem/progenitor cells were cultured in different conditions of ADSC extracellular vesicles for 7 days. As shown in Figure 4, the cartilage stem/progenitor cell proliferated with increasing culture period, and there was an obvious difference in cell proliferation between the normoxic ADSCs-derived EVs group and the hypoxic ADSCs-derived EVs group (50 μg/mL) (P < 0.05), which indicated that hypoxic ADSCs-derived EVs may promote cell proliferation.

Scratch wound assays indicated that hypoxic ADSCs-derived EVs significantly enhanced the motility of cartilage stem/progenitor cell (P < 0.05), indicating that hypoxic ADSCs-derived EVs enhanced cartilage stem/progenitor cell migration (Figure 4).

### 3.5 Gross view, SEM, and Live/dead staining

**Figure 5.**
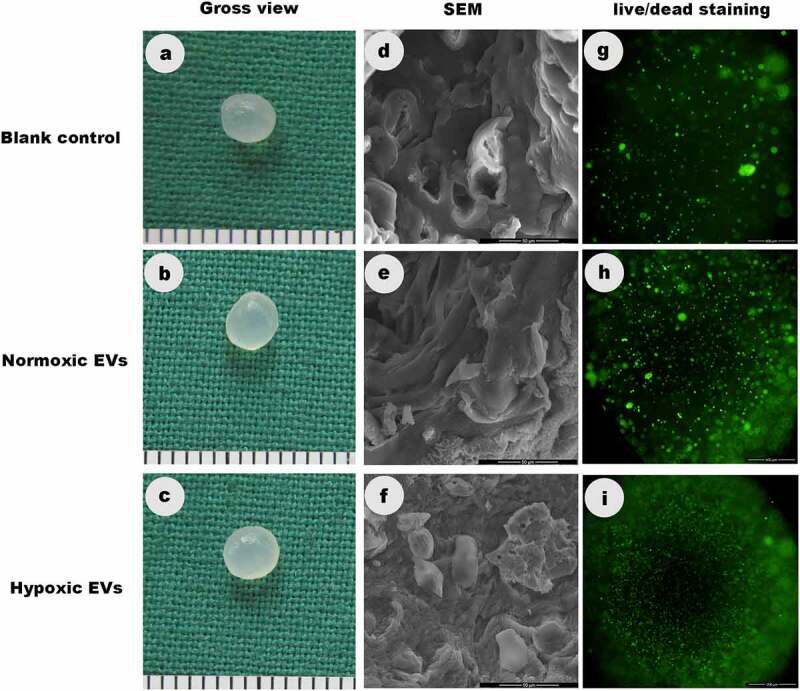
Gross view, SEM, and Live/dead staining

After 3 weeks of incubation in vitro, the constructs in three groups formed an ivory-whitish cartilage-like tissue (Figure 5a-b).

The constructs in three groups formed an ivory-whitish cartilage-like tissue after 3 weeks of incubation in vitro. SEM illustrated the cartilage stem/progenitor cell adhered, grew and spread better in hypoxic ADSCs-derived EVs group than in control groups after incubation for 3 weeks. The live/dead staining demonstrated that more cells were observed in hypoxic ADSCs-derived EVs groups than in the other two groups.

Figure 5(d-f) illustrated the morphology of the cartilage stem/progenitor cell adhering to the hydrogels after incubation for 3 weeks. It could be seen that cartilage stem/progenitor cell grew and spread better in hypoxic ADSCs-derived EVs group than in control groups.

The live/dead staining demonstrated that there were significantly more cells in hypoxic ADSCs-derived EVs groups than the other two groups. These results indicated that ADSCs-derived EVs promoted greater cell attachment and proliferation than control groups (Figure 5g-i).

### 3.6 Histological and immuno-histological staining

**Figure 6.**
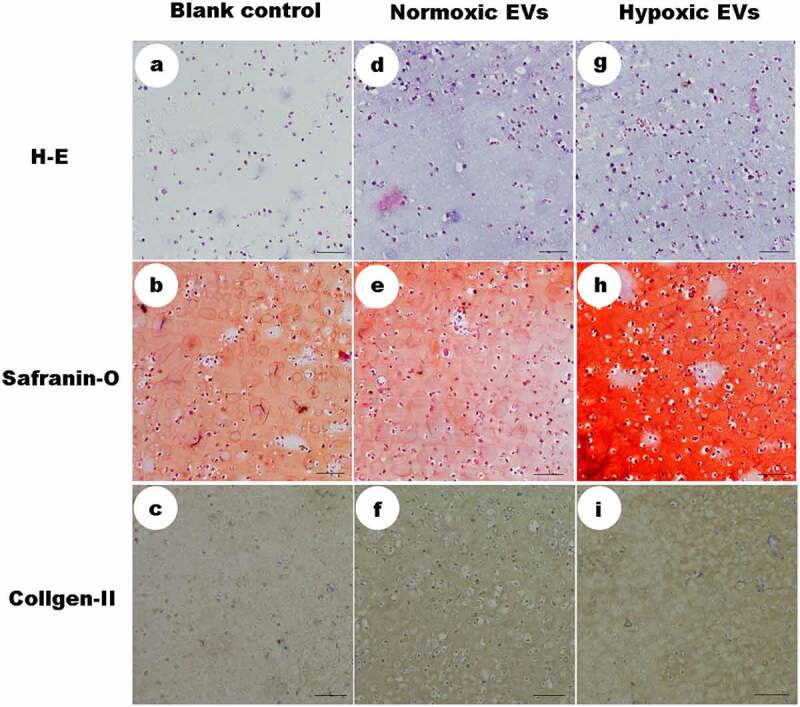
Histological and immuno-histological staining

Histological and immuno-histological staining analysis of the engineered tissue can be used to assess the formation of neo-cartilage. The histological structure of the cartilage stem/progenitor cell-hydrogel beads after 3 weeks of culture in vitro was visually observed in the HE-stained images (Figure 6). Histology further demonstrated that cartilage-like tissue was formed, with an obvious lacuna structure and stronger expression of Safranin O and type 2 collagen in hypoxic ADSCs-derived EVs group.

HE-stained images demonstrated that cartilage-like tissue was formed, with an obvious lacuna structure and stronger expression of Safranin O and type 2 collagen in hypoxic ADSCs-derived EVs group, compared to the control groups (scale = 100um).

*3.7 Hypoxic ADSCs-derived EVs promoted the chondrogenic differentiation of* cartilage stem/progenitor cell

When cultured in solution containing hypoxic ADSCs-derived EVs (50 μg/mL), the expression of aggrecan, type II collagen, and Sox-9 was significantly upregulated (p < 0.05), while the expression of these chondrocyte-specific genes in the control group remained very low, which suggested that hypoxic ADSCs-derived EVs promoted the chondrogenic differentiation of cartilage stem/progenitor cell (Figure 6).

**Figure 7.**
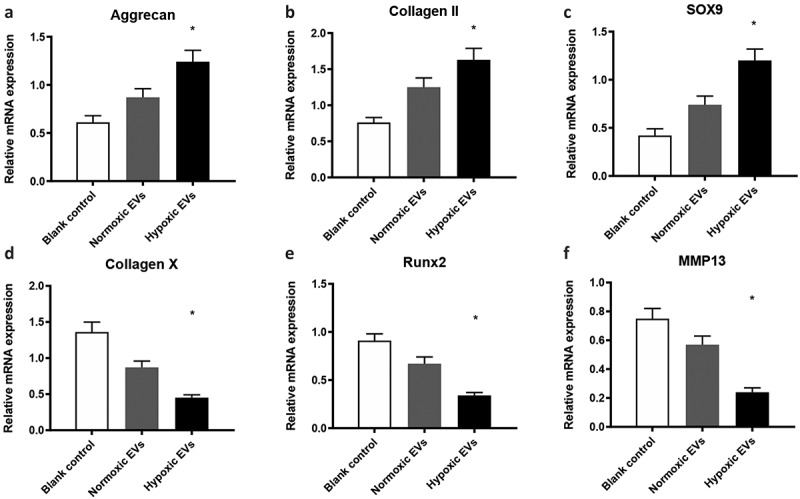
Hypoxic ADSCs-derived EVs promoted the differentiation of cartilage stem/progenitor cell

The mRNA expression of RUNX2, collagen 10a1, and MMP13 was significantly decreased when cultured in solution containing hypoxic ADSCs-derived EVs (50 μg/mL) (p < 0.05), indicating that hypoxic ADSCs-derived EVs inhibited the mRNA expression of hypertrophic genes or endochondral ossification in cartilage stem/progenitor cell (Figure 7).

The expression of these chondrocyte-specific genes (aggrecan, type II collagen, and Sox-9) cultured in solution containing 50 μg/mL was significantly upregulated (p < 0.05), while the expression of these chondrocyte-specific genes in the control groups remained very low. In addition, the RUNX2, collagen 10a1, and MMP13 mRNA expression was significantly down-regulated when cultured in solution containing hypoxic ADSCs-derived EVs (50 μg/mL) (p < 0.05)

**Figure 8.**
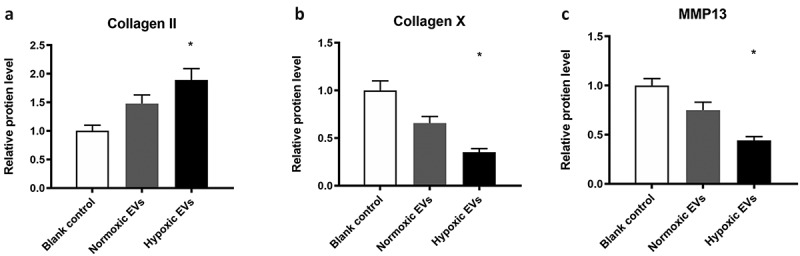
The protein levels for MMP-13, collagen II and collagen X

In addition, the hypoxic ADSCs-derived EVs down-regulated the protein expression of MMP-13, collagen II and collagen X of the cell-hydrogel construct significantly (50 μg/mL) (p < 0.05) (Figure 8).

The hypoxic ADSCs-derived EVs down-regulated the protein expression of MMP-13, collagen II and collagen X of the cell-hydrogel construct significantly (p < 0.05).

### 3.8 Quantitative analysis of in vitro cartilage formation

Matrix synthesis contributes greatly to the mechanical strength of cartilage stem/progenitor cell-hydrogel constructs, and glycosaminoglycan (GAG) content and total collagen content were analysed in the current study.

**Figure 9.**
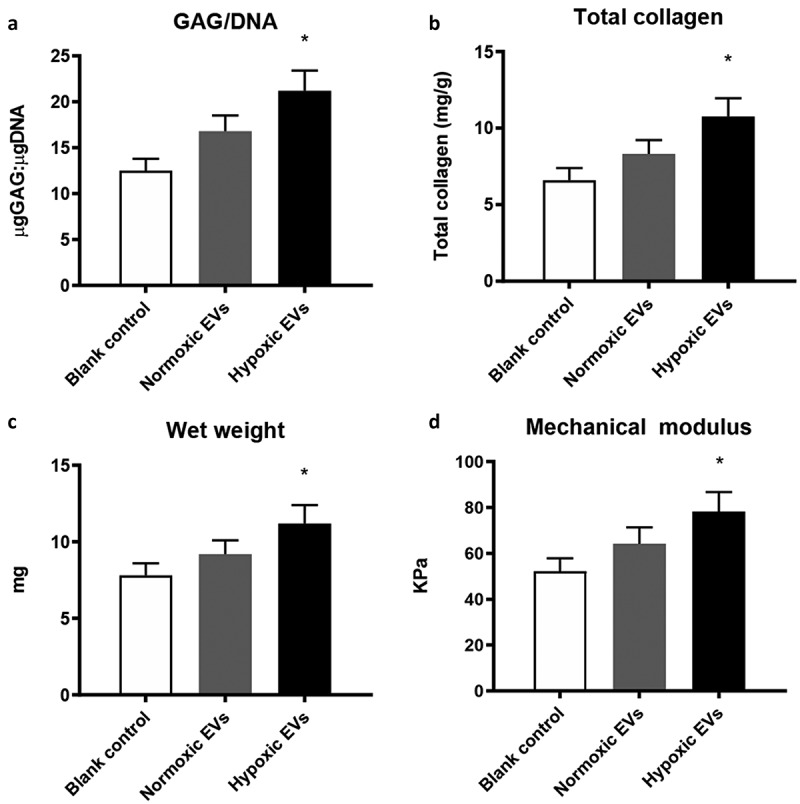
Quantitative analysis of in vitro cartilage formation

There was a significant difference between the hypoxic group and the control groups (p < 0.05). A significant increase in GAG content with incubation time was detected, while a significant difference in GAG content among the three groups was observed on day 21. The integration of hypoxia mediated by ADSC-derived EVs with alginate gel significantly induced more cartilage matrix and proteoglycan production (p < 0.05) (Figure 9).

The integration of hypoxia mediated by ADSC-derived EVs with alginate gel significantly induced more cartilage matrix and proteoglycan production (p < 0.05). Hypoxic ADSCs-derived EVs enhanced the compressive strength of the cartilage stem/progenitor cell-hydrogel composite, there is a significant difference among three groups (p < 0.05).

### 3.9 *Hypoxic ADSCs-derived EVs enhanced the compressive strength of the* cartilage stem/progenitor cell*-hydrogel composite*

As shown in Figure 8, the compressive strength measurement showed 52.24 ± 5.65 KPa at 3 weeks in the control group. The compressive strength of cartilage stem/progenitor cell-hydrogel composite in normoxic group was 64.18 ± 7.23 KPa at 3 weeks, while the compressive strength of the cartilage stem/progenitor cell-hydrogel composite in hypoxic was 78.33 ± 8.45 KPa at 3 weeks. The compressive modulus significantly differed among the three groups, which indicated that hypoxic ADSCs-derived EVs contributed to the stable mechanical strength of the cartilage stem/progenitor cell-hydrogel constructs (p < 0.05) (Figure 9).

## 4. Discussion

Cartilage defects remain a major clinical challenge, as cartilage has very low proliferation and self-healing ability. Full-thickness cartilage lesions are more common in athletes than in the general population; 36% of 931 athletes had full-thickness lesions on MRI, but only 14% of these cartilage lesions presented with associated symptoms [42]. Stem cell-based treatments provide early favourable clinical results, while they result in fibrocartilage formation growth and excessive ossification with poor biomechanical properties over time [43]. Chondrocytes or cartilage-based therapy promote hyaline-like cartilage formation, such as osteochondral autografting and chondrocyte transplantation, which can restore normal biomechanical characteristics and prevent fibrocartilage growth and endochondral ossification [44,45]. Chondrocyte-based regenerative medicine for treating cartilage defects is favourable, although it requires sufficient expansion of differentiated autologous chondrocytes. The main drawbacks are limited chondrocyte availability and dedifferentiation of chondrocytes after long-term culture.

In our previous study, we isolated cartilage stem/progenitor cells from cartilage tissue by a fibronectin differential adhesion assay, and the cell populations expressed MSC surface markers, as indicated by flow cytometric analysis, and these cells could differentiate into osteogenic, chondrogenic and adipogenic lines under different induction conditions [19,20]. And cartilage stem/progenitor cells possessed an inherent chondrogenic advantage, indicating that cartilage stem/progenitor cells are a promising cell source for cartilage regenerative medicine application.

Hypoxia has also been reported to enhance the proliferation and chondrogenic potential of chondrocytes and mesenchymal stromal cells [46–48]. Wan found that hypoxia enhanced the viability, growth and chondrogenic potential of cryopreserved human adipose-derived stem cells [49]. In current study, the effect of hypoxia on cartilage stem/progenitor cells was evaluated, and we found that hypoxia promoted the proliferation and migration of cartilage stem/progenitor cells. In addition, hypoxia enhances the chondrogenic potential of cartilage stem/progenitor cells by up-regulating the expression of sox-9, collagen-II, and aggrecan, and inhibits the expression of collagen-X, RUNX2, and MMP13. Lee et al. that found in their study the PI3K/Akt/FoxO survival pathway activated by hypoxia in MSCs enhances chondrogenesis and plays an important role in preventing endochondral ossification [50].

It has also been reported that ADSCs promoted the proliferation of articular cartilage chondrocytes when cocultured with chondrocytes [51], which may be because ADSCs secrete certain factors with chondro-protective effects, such as chondrocyte proliferation and cartilage matrix protection [52]. Recent studies have demonstrated that MSCs exerted their therapeutic effects by secreting soluble or ‘paracrine’ factors, known as extracellular vesicles [53]. Therefore, the combination of hypoxia and ADSCs-derived EVs may promote the proliferation and differentiation of cartilage stem/progenitor cells in vitro.

In the current study, we focused on the effect of hypoxic ADSCs-derived EVs on cartilage stem/progenitor cells proliferation and activity. ADSCs isolated from adipose tissue can differentiate into multiple cell types (adipocytes, osteoblasts, and chondrocytes), which show great therapeutic potential in regenerative medicine and tissue engineering. It has been shown that the biological effects of ADSCs depend mainly on paracrine action via secreting extracellular vesicles [54]. Compared with stem cells of other origins, ADSCs have the distinct advantages of minimal invasivity and greater supply and harvesting. In addition, the paracrine characteristics of ADSCs demonstrated more anti-inflammatory and immune-modulating effects, which differed from bone marrow stem cells [55].

In the current study, extracellular vesicles were isolated and purified from ADSCs under normoxia and hypoxia condition. The results indicated that the ADSC-derived EVs ranged mainly from 40 nm and 1000 nm in diameter and expressed exosome markers, including CD9, CD63, and CD81, which plays an important role in intercellular communication, allowing extracellular vesicles to serve as vehicles for the transfer of membrane and cytosolic proteins, lipids, and RNA between cells. It has been demonstrated that extracellular vesicles have common surface markers, such as CD9, CD63, and CD81 [56].

In the current study, the effects of extracellular vesicles on cartilage stem/progenitor cells proliferation and migration were evaluated, and the results indicated that normoxic ADSCs extracellular vesicles and hypoxic ADSC-derived EVs significantly promoted cartilage stem/progenitor cell proliferation and migration, which can be explained by the fact that MSC extracellular vesicles activated several signalling pathways (Akt, ERK, and STAT3) and induced the expression of a number of growth factors (HGF, IGF1, NGF and SDF1), which may enhance proliferation and migration [57]. Some studies have demonstrated that stem cell-based tissue repair and regeneration depend on paracrine mechanisms mediated by extracellular vesicles and that extracellular vesicles could induce cell proliferation and migration [58]. Shabbir A et al. found that extracellular vesicles isolated from human umbilical vein endothelial cells led to in a dose-dependent enhancement of proliferation and migration of fibroblasts derived from normal donors and chronic wound patients [57]. In another study, Wang et al. demonstrated the involvement of extracellular vesicles-mediated communication in bone marrow stem cell (BMSC)-induced proliferation, migration, survival, and drug resistance of multiple myeloma cells [59].

In addition to enhancing cartilage stem/progenitor cells proliferation and migration, ADSC-derived EVs enhanced the differentiation of cartilage stem/progenitor cells, as the expression of GAGs, ACAN and COL2A1 were enhanced. The expression of GAGs, ACAN and COL2A1 was increased in chondrogenic differentiation processes, while the expression of hypertrophic markers (COL10A1, ALP, RUNX2) was simultaneously increased. In recent decades, multiple methods have been developed to inhibit chondrocyte terminal differentiation [12]. Caron et al. reported that 3D cultures exhibited the most potent chondrogenic potential for redifferentiation of dedifferentiated chondrocytes, whereas a hypertrophic phenotype was best achieved in 2D culture [60]. In the current study, we found hypoxic ADSCs-derived EVs up-regulated the expression of GAGs, ACAN and COL2A1 in chondrogenic differentiation processes, while it inhibited the expression of hypertrophic markers (COL10A1, ALP, RUNX2), compared to the control groups.

For 3D culture, the integration of ADSC-derived EVs with alginate gel was used to evaluate the effect of ADSC-derived EVs on cartilage stem/progenitor cells, and cell proliferation and matrix synthesis contributed to the increased mechanical characteristics observed in the current study. The results of histological staining, biochemical and biomechanical analysis of the hypoxic ADSC-derived EVs group were superior to those of the control groups, suggesting that hypoxic ADSC-derived EVs promoted the formation of cartilage stem/progenitor cells-alginate gel constructs.

However, there are some limitations in this study that could be addressed in future research. First, more data analysis should be performed to support the statements of this study; Second, in vivo animal study should be performed to demonstrate superior cartilage repair or formation of a more phenotypically stable cartilage with the use of hypoxic EVs. And there may be some other possible limitations in this study.

## Conclusions

In summary, our study found that hypoxic ADSC-derived EVs not only could promote the proliferation and viability of cartilage stem/progenitor cells but could also increase chondrogenic differentiation and inhibit the expression of chondrocytes hypertrophic gene.

## Supplementary Material

Supplemental Material

## Data Availability

All data generated or analyzed during this study are included in this published article. For additional information, please contact the author.

## Abbreviations

Cartilage stem/progenitor cells (CSPCs); extracellular vesicles (EVs); hypoxic adipose-derived stem cells (ADSCs); Mesenchymal stromal cells (MSCs); COL II: Collagen type II; COL X: Collagen type X; CPCs: Cartilage progenitor/stem cells; DMEM: Dulbecco’s modified Eagle’s medium; FBS: Foetal bovine serum; H&E: Haematoxylin and eosin;

MMP 13: Matrix metalloproteinase 13; PBS: Phosphate-buffered saline; RT-PCR: Real-time quantitative polymerase chain reaction; TEM: Transmission electron microscopy; TGF-β: Transforming growth factor-β
