# Supplementary figures and images for: Hypoxic ADSCs-derived EVs promote the proliferation and chondrogenic differentiation of cartilage stem/progenitor cells
Source: Adipocyte. 2021 Jul 5;10(1):322–37. doi: 10.1080/21623945.2021.1945210 (PMC8259721; doi:10.1080/21623945.2021.1945210)

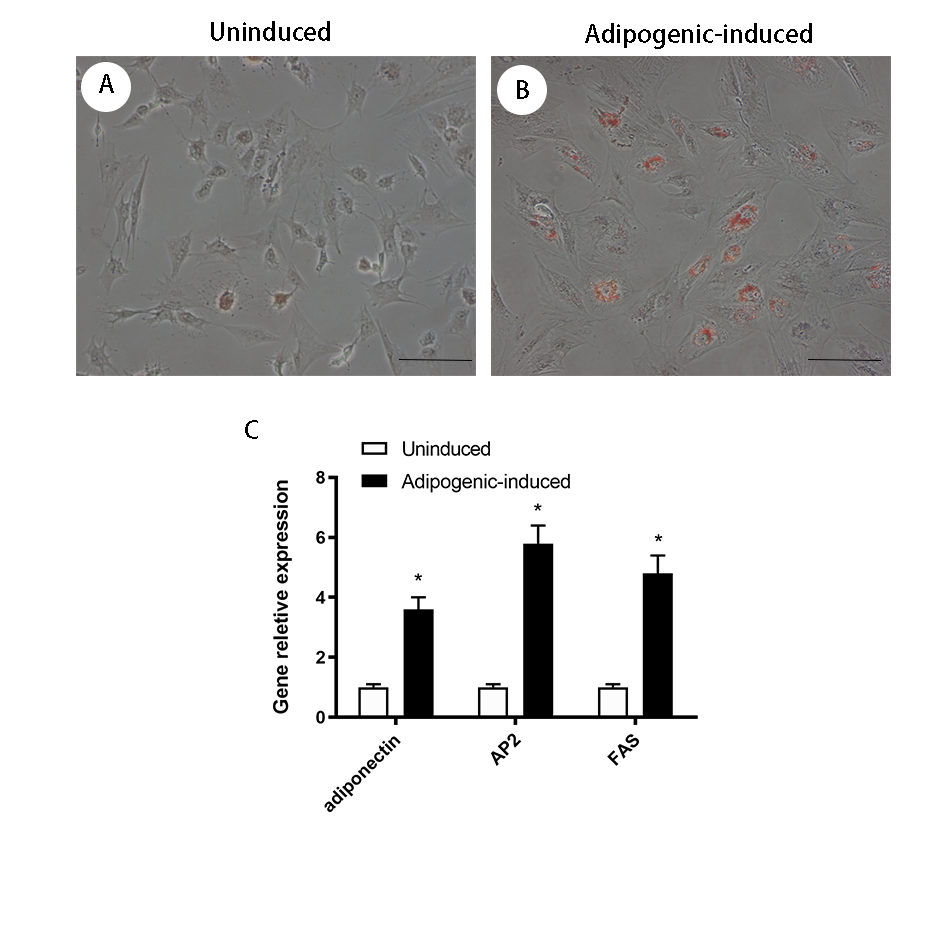

Supplement: Supplemental Material [file KADI_A_1945210_SM6570.zip › supplementary/Figure S1.tif]

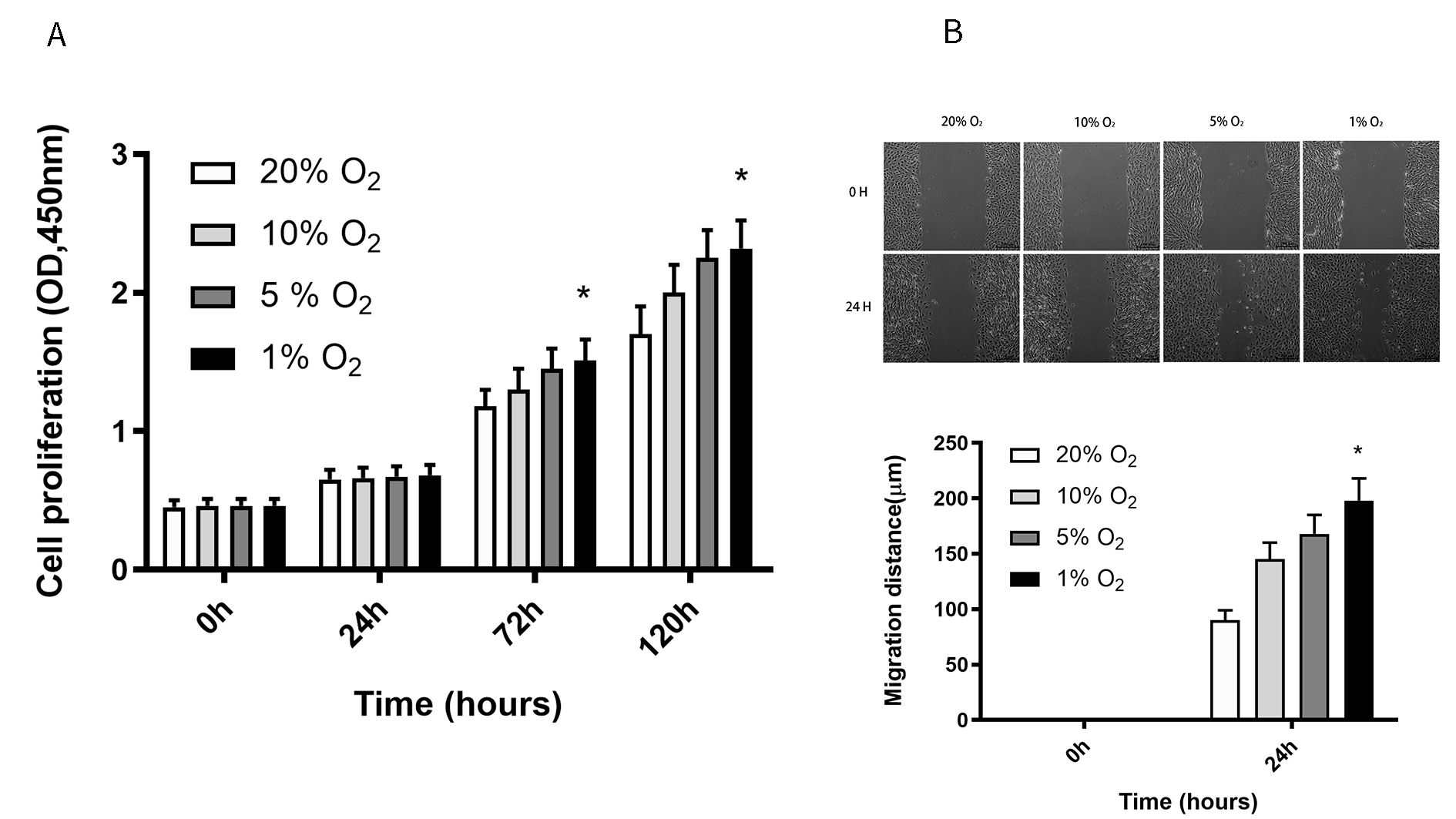

Supplement: Supplemental Material [file KADI_A_1945210_SM6570.zip › supplementary/Figure S2.tif]

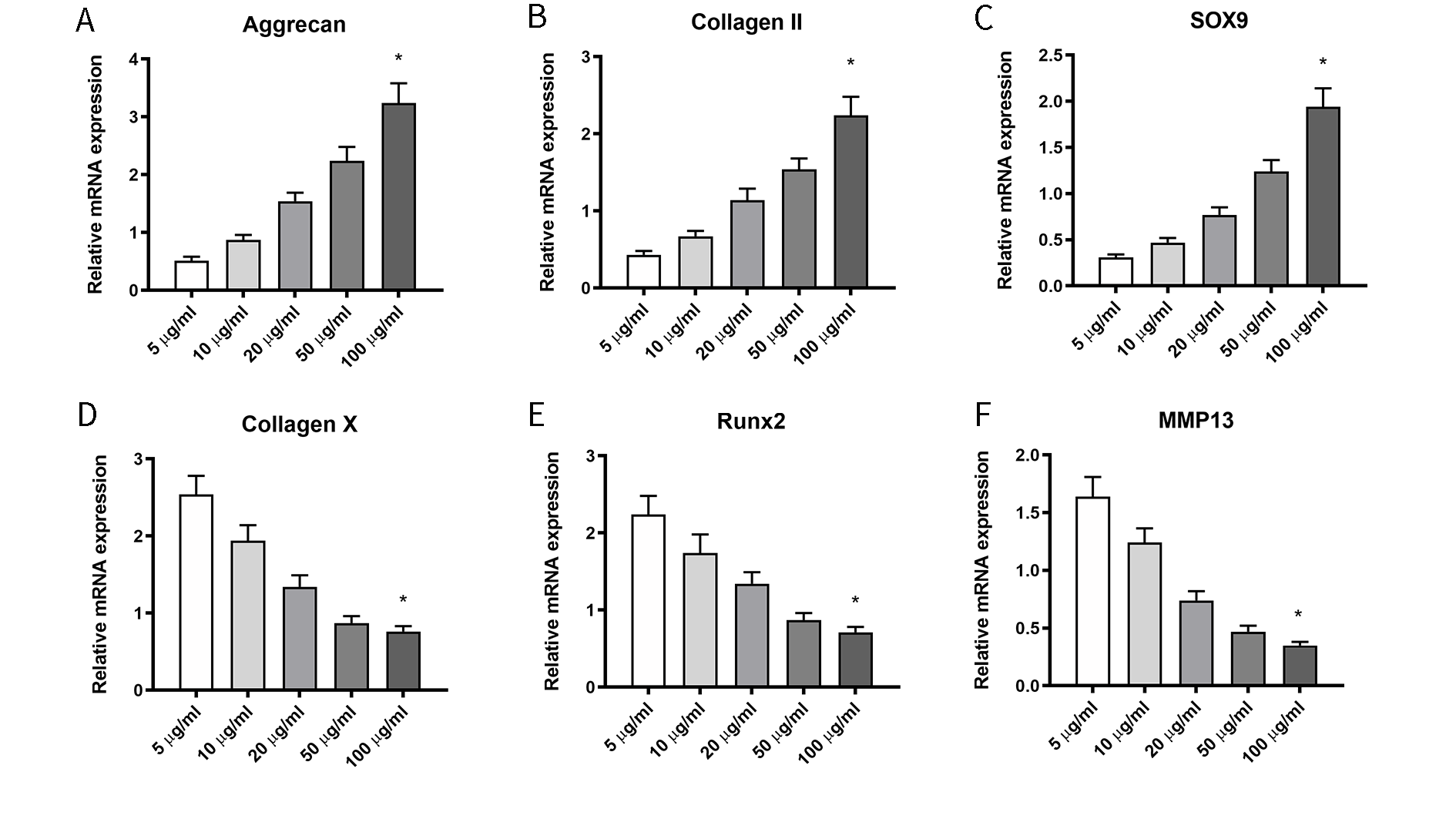

Supplement: Supplemental Material [file KADI_A_1945210_SM6570.zip › supplementary/Figure S3.tif]
